# A Case of Sjögren's Syndrome Associated With Trigeminal Neuropathy and Enhancement of the Mandibular Nerve at the Foramen Ovale: A Case Report and a Review of the Differential Diagnosis and Mechanisms of the Disease

**DOI:** 10.7759/cureus.19463

**Published:** 2021-11-11

**Authors:** Mohamed Ziad Farran, Hassan Kesserwani

**Affiliations:** 1 Neurology, Trinity College, Dublin, IRL; 2 Neurology, Flowers Medical Group, Dothan, USA

**Keywords:** gasserian ganglion, meckels cave, foreamen ovale, enhanced mandibular nerve, neuropathy, trigeminal neuropathy, sjogren syndrome

## Abstract

We describe a case of Sjögren's syndrome-associated trigeminal neuropathy with mandibular nerve enhancement at the foramen ovale and Gasserian ganglion (Meckel's cave) in a patient with a prior history of breast cancer. We also explore the differential diagnosis of trigeminal neuropathy associated with mandibular nerve involvement at the foramen ovale and exclude other diseases such as Sjögren's syndrome or perineurial invasion as a result of various carcinomas. We emphasize the importance of an investigative triad of searching for a local head-and-neck malignancy that may metastasize by perineural spread or invasion, excluding a distant malignancy or paraneoplastic phenomenon and ruling out an autoimmune etiology such as Sjögren's syndrome. In the process, we briefly outline the basic immunopathologic processes.

## Introduction

The trigeminal nerve, the fifth cranial nerve, is the largest of the cranial nerves. It is derived embryologically from the first pharyngeal arch and possesses both motor and sensory functions. The motor branch directly transmits from the motor nucleus of the trigeminal nerve to the muscles of the face. On the other hand, the sensory branch has a circuitous course emanating from the mesencephalic nucleus, principal sensory nucleus, and the spinal nuclei of the lower medulla and upper cervical cord. The axons then relay to the contralateral thalamus and to the post-central gyrus of the cerebral cortex, conveying sensory information (touch, pressure, and pin-prick) [1,2].

The trigeminal nerve gives rise to three branches: the ophthalmic nerve (V1), the maxillary nerve (V2), and the mandibular nerve (V3). The ophthalmic and maxillary nerves are predominantly sensory whereas the mandibular nerve is responsible for both sensory and motor information. The ophthalmic nerve provides sensory innervation to the face at a level above the orbits, the superior portion of the nasal cavity, the frontal sinus, the dura mater, and portions of the anterior cranial fossa. In addition to this, the ophthalmic nerve also innervates the ciliary body, iris, lacrimal gland, conjunctiva, and cornea and is responsible for pupillary dilation. Critically, trigeminal V1 traverses the cavernous sinus when it exits the superior orbital fissure to become the supraorbital nerve. A trigeminal nerve deficit and a sixth nerve palsy may localize a lesion to the cavernous sinus [3]. The maxillary nerve supplies the region between the orbit and the mouth, which includes the inferior portion of the nasal cavity and the maxillary teeth. The maxillary nerve also enters the cavernous sinus and exits the base of the skull via the foramen rotundum to enter the pterygopalatine fossa. The mandibular nerve, on the other hand, provides sensory information to the mandibular teeth, buccal mucosa, temporomandibular joint, the anterior two-thirds of the tongue, and the face below the territory of the maxillary nerve. It also provides motor innervation to the muscles of mastication. The mandibular nerve passes through the foramen ovale, at the base of the skull before it enters the mandibular foramen at the medial surface of the ramus, transverses through the mandibular canal, and then exits the mandible anteroinferiorly via the mental foramen as the mental nerve [4]. A mental nerve neuropathy may be a sinister sign of a metastatic malignancy [5]. The mandibular nerve also supplies the tensor veli palatini and tensor tympani, whose impairment can lead to palatal myoclonus and hyperacusis, respectively [6].

The trigeminal nerve is subject to neuropathic processes including local malignant perineural invasion, autoimmune processes such as Sjögren's syndrome and mixed connective tissue disease, paraneoplastic processes, or it can be idiopathic [7-9]. Trigeminal neuropathy usually manifests as numbness or tingling in the regions that are innervated by the trigeminal nerve, the surface of the skin, or more deeply in the mucosal regions. Malignancies, in particular, are extremely important to be considered, as they may indicate the first manifestation of the disease or an indication of relapse in patients with a prior history of a malignant process. In such cases, it is pivotal to exclude any abnormality at the skull base or within the proximity of the trigeminal ganglion. This may be overlooked especially when the numbness spreads gradually or the trigeminal motor function and corneal reflexes remain intact [10]. Furthermore, it is important that all cases of trigeminal neuropathy workup include an assessment of the skull base with a high-resolution MRI study with and without contrast enhancement.

## Case presentation

A 76-year-old female presented to the clinic with a several-month history of numbness in the left half of her face - forehead, cheek, and chin - accompanied by sharp shooting pain. It was also characterized by a prominent lack of sensation of the left upper and lower gums and the inside of the cheek pouch, without inadvertent painless cuts. Taste and salivation were unaffected. She also complained of a dry and itchy left eye, which had been treated with saline eye drops, and an unintentional weight loss of 10 pounds over the past year. There were no complaints related to vision, swallowing or speech difficulty, or acral numbness.

Her past medical history was significant for a two-year history of idiopathic polyarthritis treated successfully with hydroxychloroquine 200 mg twice daily. Additionally, she had a history of breast cancer requiring a lumpectomy and six cycles of chemotherapy with Taxotere and cyclophosphamide, completed in 2012, followed by radiation therapy. She was noted to be in remission with regular screening for malignant disease, with a recent negative positron emission tomography (PET) scan. She was on maintenance tamoxifen therapy 20 mg daily. Her other medications included vitamin D supplementation and once-monthly intramuscular injections of 1-mg vitamin B12. She had no history of smoking and denied any fatigue, headaches, shortness of breath, or other constitutional symptoms.

She was 5 feet 6 inches tall, weighed 114.1 pounds, with a body mass index of 18.4 kg/m^2^. Her vitals revealed a blood pressure of 172/90 mmHg, a heart rate of 63 beats per minute, and oxygen saturation of 98%. On physical examination, the patient appeared thin but well-nourished and in no acute distress. She was oriented to person, place, problem, and time with appropriate mood and affect. Gait stance and cadence were normal. Tandem walking was normal and the Romberg sign was absent.

The cranial examination revealed hypesthesia to touch in the V1, V2, and V3 distribution of the left trigeminal nerve. The corneal reflex was preserved to touch. The masseter function was also preserved with clenching of the teeth, but there was bilateral wasting of the temporal fossae. Motor function of the left seventh cranial nerve was entirely normal to forehead elevation, smiling, and blowing of the cheeks. Pterygoid function with jaw deviation and masseter function with teeth clenching were also normal. The rest of the cranial nerve examination was normal.

Motor function of the upper and lower extremities revealed normal muscle bulk and tone, and a 5/5 motor response on the Medical Research Council (MRC) rating scale. The handgrip was symmetric without pronator drift. Skeletal examination of the joints, bones, and muscles showed no signs of contractures, malalignment, tenderness, or bony abnormalities and there was a normal range of all extremities. Deep tendon reflexes were intact in both upper extremities. Deep tendon reflexes at the ankles bilaterally were absent. No spastic catch was noted in the arms and the plantar Babinski sign was absent bilaterally. Coordination with finger-to-nose and heel-to-shin was normal bilaterally. Sensory examination was normal to touch-pressure, vibration, and joint-position-sense in the fingers and toes.

**Figure 1:**
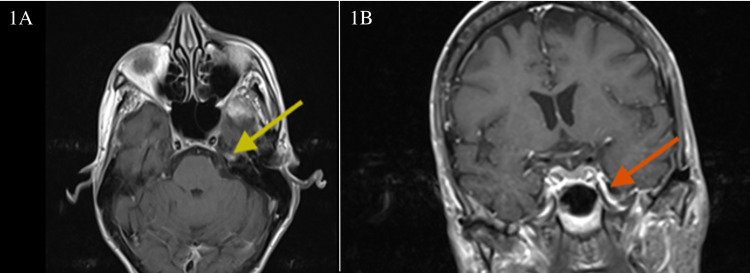
T1 post-contrast weighted MRI images of the brain 1A: Axial T1 post-contrast weighted MRI images with gadolinium enhancement of the semilunate Gasserian ganglion (yellow arrow). 1B: Coronal T1 post-contrast weighted MRI images demonstrating mandibular nerve enhancement in the foramen ovale (red arrow) MRI: magnetic resonance imaging

An MRI of the brain with and without contrast revealed an 11-mm enhancement of the left mandibular nerve within the left foramen ovale on T1-weighted sequences (Figure 1).

A lumbar puncture with cerebrospinal fluid (CSF) analysis was performed and the findings are summarized below (Table 1).

**Table 1:** Results of CSF analysis CSF: cerebrospinal fluid; N/A: not applicable; IgG: immunoglobulin G

Test	Result	Interpretation	Reference range	Units
IgG, quantitative	7.0	Above high normal	0.0-6.7	mg/dL
Albumin	21	Normal	10-46	mg/dL
IgG, serum	2546	Above high normal	586-1602	mg/dL
Albumin	3.6	Below low normal	3.7-4.7	g/dL
IgG/albumin ratio	0.33	Above high normal	0.00-0.25	N/A
IgG index	0.5	Normal	0.0-0.7	N/A
Oligoclonal bands	4 bands	Normal	Less than 4 bands	N/A

Oligoclonal banding testing was performed by using isoelectric focusing (IEE) and immunoblotting. Zero oligoclonal bands were observed in the CSF. However, four paired bands were observed in both the CSF and serum. Paired bands suggest an immune response to an inflammatory process outside the central nervous system (CNS) and are unlikely to represent a CNS inflammatory disease.

Sjögren’s syndrome serology testing revealed findings that are summarized below (Table 2).

**Table 2:** Sjögren’s syndrome serology SS: Sjögren's syndrome; N/A: not applicable

Test	Result	Interpretation	Reference range	Units
Sjögren’s anti-SS-A	8.0	Above high normal	0.0-0.9	N/A
Sjögren’s anti-SS-B	<0.2	Normal	0.0-0.9	N/A

Furthermore, a serum paraneoplastic panel including anti-acetylcholine receptor ganglionic neuronal antibodies, anti-amphiphysin antibodies, anti-glial nuclear antibody type 1, anti-neuronal nuclear antibody type 2, anti-neuronal nuclear antibody type 3, collapsin response-mediator protein-5 (CRMP-5) immunoglobulin G, neuronal voltage-gated potassium channel antibodies, calcium channel antibody P/Q-type and Purkinje cell cytoplasmic antibodies were all found to be negative. An ear-nose-throat (ENT) referral and evaluation revealed no cervical adenopathy or neck masses.

Our provisional diagnosis was Sjögren's syndrome-associated trigeminal neuropathy based on the sicca symptoms of dry eyes, the positive serology for Sjögren's syndrome, trigeminal ganglion and mandibular nerve enhancement, and prior history of hydroxychloroquine-responsive polyarthritis. Significantly, a PET scan was negative for metastatic disease given her prior history of breast cancer. Furthermore, an ENT evaluation precluded the presence of a head-and-neck malignancy. Interestingly, CSF analysis revealed four oligoclonal bands in both serum and CSF, implying a systemic autoimmune disease, supporting the diagnosis of Sjögren's syndrome. The patient was given a titrating dose of prednisone at 0.5 mg/kg for one month without relief of facial numbness and dysesthesias. A course of duloxetine at 60 mg a day improved the neuropathic symptoms of dysesthesia and sharp tingling pain.

## Discussion

Sjögren's syndrome is an autoimmune system disease with a predilection for the lacrimal and salivary glands and their innervating fibers, leading to its cardinal manifestations of dry eyes and dry mouth. Polyarthritis is quite common and is usually responsive to hydroxychloroquine. Renal involvement is rare and, when present, may lead to a poor prognosis. Sjögren's syndrome represents a risk factor for progression to non-Hodgkin's lymphoma. Immunologically, there is upregulation of toll-like receptors (TLR) that recognize viral epitopes in target organs and the elaboration of interferon-gamma and its adverse effects of inflammatory cell recruitment. Positive serology to SS-A, SS-B, anti-salivary gland protein-1 (SP1), anti-carbonic anhydrase-6 (CA6) enzyme, and anti-parotid secretory protein (PSP) is highly variable. A definitive diagnosis is made by correlating the clinical history, serology, and a confirmatory salivary gland biopsy [11].

Neurologic involvement in Sjögren's syndrome is fascinating. The most common manifestation is painful small fiber neuropathy, which is usually a low-grade microvasculitis. The latter can also cause a mononeuritis multiplex, which is far less common. One of the classic presentations is a polyganglioneuropathy presenting as sensory ataxia, which is due to the inflammation of the dorsal root ganglia. Therapeutic response to steroid therapy and intravenous immunoglobulin is highly variable. Trigeminal nerve involvement can occur in about one-fifth of cases and can be unilateral or bilateral, and is usually inflammatory as in ganglioneuropathy [12]. A co-existent painful vasculitic sensory neuropathy is not uncommon, implying a vasculitic trigeminal neuropathy as another pathogenic mechanism [13]. Involvement of the trigeminal nerve may present with frank sensory loss or pain. The latter can be due to dysesthesias, crawly uncomfortable sensations, or a bona fide trigeminal neuralgia-like pain. A useful diagnostic clue as to the site of inflammation is the involvement of the mandibular nerve (V3); the latter can be solitary or involve the V2 and V3 branches implicating the Gasserian trigeminal ganglion, indicating that the mandibular branch bypasses the cavernous sinus when it enters the Gasserian ganglion [14]. Trigeminal neuropathy arising from metastatic breast cancer is extremely uncommon and has rarely been documented [15].

The cranial nerves are surrounded by a perineural vascular plexus. MRI enhancement of the trigeminal Gasserian ganglion and the mandibular nerve (V3) proper are very uncommon, occurring in 4% and 3% of cases respectively. This should not be confused with perineural vascular plexus enhancement [16]. One needs to distinguish between perineural spread and perineural invasion. The former is a purely radiological diagnosis that refers to malignant spread along the nerve visible on imaging studies and the latter is a pathological diagnosis made on biopsy, which is defined histologically as the presence of malignant cells in the perineural space. The two can be hard to differentiate. Understanding perineural spread and invasion is also quite complicated. Of note, the cranial nerves do not harbor lymphatic channels and the surrounding collagenous complex of the perineurium provides a rather unbreakable barrier [17]. An interesting anatomical observation is that the trigeminal nerve (V) and the facial nerve (VII) are the most commonly involved nerves with malignant perineural invasion and autoimmune inflammatory processes. There are interesting anatomical connections between these two cranial nerves. The first is an anastomosis mediated by the greater superficial petrosal nerve (GSPN) via the pterygoid canal, linking the geniculate ganglion (cranial nerve VII) and the pterygopalatine ganglion (V2). This can explain the symptom complex of loss of taste sensation, hyperacusis, loss of sensation of ear structures, and absence of tearing. The second anastomosis is between the auriculotemporal nerve (V3) and the lesser superficial petrosal nerve (VII) near the stylomastoid foramen. This explains the symptom complex of anesthesia of the ear structures, temporal region, and lack of salivation. These anastomotic connections are also thought to provide a conduit for perineural spread.

Invasion of this collagenous wall in the perineurium involves a unique cascade of events. This may involve the release of chemokines and attraction of chemokine-receptor positive tumor cells and the release of proteinases such as matrix metalloproteinases by malignant cells, allowing the breakdown of the collagenous matrix barrier. The histologic subtype of the tumor is also relevant as this may involve the release of growth factors such as glia-derived nerve factor (GDNF) and nerve growth factor (NGF). The expression of neural cell adhesion molecule (NCAM) also allows for perineural spread and the salivary adenoid cystic carcinoma has the highest propensity for perineural invasion [18,19]. Perineural invasion of the mandibular nerve by head-and-neck malignancies is quite common with squamous cell carcinoma, adenoid cystic carcinoma, salivary duct carcinoma, and polymorphous low-grade adenocarcinoma [20].

## Conclusions

The trigeminal nerve has a fascinating and circuitous route from the brainstem to the periphery with multiple sensory, motor, and autonomic functions. Its clinical manifestations are relatively obvious and should alert one to a definite and small set of differential diagnoses. This article highlights the critical importance of getting an MRI of the base of the brain in diseases of the trigeminal system; moreover, correlation with the clinical symptomatology is paramount. Furthermore, understanding skull base anatomy and the pathophysiologic basis of disease will help the clinician diagnose and treat patients in an accurate and better manner.
